# Editorial: Role of extracellular matrix in neurodevelopment and neurodegeneration

**DOI:** 10.3389/fncel.2023.1135555

**Published:** 2023-01-18

**Authors:** Vishwa Mohan, Chandrakanth Reddy Edamakanti, Amrita Pathak

**Affiliations:** ^1^Davee Department of Neurology, Northwestern University Feinberg School of Medicine, Chicago, IL, United States; ^2^Annexon Biosciences, Brisbane, CA, United States; ^3^Department of Biochemistry, Vanderbilt Brain Institute, Vanderbilt University School of Medicine, Nashville, TN, United States

**Keywords:** extracellular matrix, perineuronal nets, neurodegeneration, neurodevelopmental disorders, proteoglycans

Extracellular matrix (ECM) is a dense and dynamic network of proteins and sugars embedding various types of cells of the nervous system. It is composed of numerous macromolecules like collagen, elastin, fibronectin, laminin, glycoproteins like tenascin, glycosaminoglycans (GAGs), and proteoglycans. These components are secreted by both neurons and glial cells. It constitutes around 20% of brain volume, yet it has not received the required attention from neuroscience research community. So far, a majority of research focus has been on either neuron or glial cellular components. The role of extracellular system on the etiology and progression of brain disorders and vice versa, how neurological disorders affect the extracellular matrix remains largely unexplored.

The ECM is known to play multiple roles during neurodevelopment, however its role in development of human brain is not fully understood. Condensed ECM, comprised of the perineuronal net (PNN), forms a mesh-like structure surrounding the cell body and proximal neurites of neurons (Sigal et al., 2019). During nervous system development ECM modulates neural progenitor cell proliferation and differentiation. It also governs the cellular morphology including axonal and dendritic elongation regulating their connectivity and cortical folding. Additionally, ECM stores signaling factors that create a microdomain to regulate neuronal migration and synaptic plasticity (Dityatev et al., 2010; Dick et al., 2013). The PNN is thought to act as a molecular brake to close and regulate the critical period of synaptic plasticity (Dityatev et al., 2010; Wang and Fawcett, 2012). Thus, ECM dysfunction, especially PNN impairment has been linked to several neurodevelopmental disorders like autism spectrum disorders, schizophrenia, bipolar disorder, Fragile X syndrome and epilepsy (Reinhard et al., 2015; Rogers et al., 2018; Wen et al., 2018).

Several decades of research on neurodegenerative diseases has indicated increased neuron death but the mechanism behind the ill health of neurons is still far from clear. The function and capabilities of extra cellular matrix around the dying cell has not been investigated in detail. Recently, an interplay between neurodegeneration, extra cellular space and matrix is reported in Parkinson's disease rodent model shedding light upon a neglected compartment for the diffusion of aggregated α-synuclein seeds (Soria et al., 2020). As reviewed recently by Pinter and Alpar, selective ECM components can either proactively trigger the disease-specific toxicants, or reactively accumulate them in ECM (Pinter and Alpar, 2022). Several studies have linked ECM to neurodegeneration in Alzheimer's, Parkinson's, amyotrophic lateral sclerosis (ALS) and Huntington diseases but there are still huge knowledge gaps regarding the role of ECM and therapeutic potential of its components.

This special issue comprises a collection of original research and review articles which enhance our understanding on the diverse role of ECM in brain development and neurodegeneration.

A well-illustrated article by Long and Huttner reviewed the role of ECM in human neocortex development and compared it to non-human primates. They focused on the neurodevelopmental processes like neural progenitor proliferation and differentiation, elongation and connectivity of neurites, neuronal migration, cortical folding, and neurodevelopmental disorders. With long lasting molecular composition and highly robust nature, ECM is often seen as a stable structure required to maintain neural network in shape. However, in order to support synaptic changes in the adult brain, ECM has the abilities to be remodeled at synapses. ECM remodeling includes regulated secretion of proteolytic enzymes at the synapse along with synthesis of new ECM molecules. Using beautiful figure depictions Dankovich and Rizzoli wonderfully reviewed the existing paradigm for ECM remodeling and recycling that allows synapse to be plastic. The review article by Chen et al. has discussed how sensory activities impact multiple neuronal and glial structures along with the extracellular components within the brain. Sensory deprivation has been shown to impact dendrites and their associated spines, particularly focusing on the cerebral cortex using the rodent whisker-to-barrel system as an illustrative model. This review provided a better understanding of structural plasticity, encompassing multiple aspects of neuro-glial cells, and extra-cellular domain interactions as a system. ECM is required to be remodeled for efficient synaptic plasticity and brain functioning.

Mood disorders and anxiety resulting from environmental stress is a big concern for all of us nowadays, and have caught the interest of Laham and Gould as well. They have contributed a mini-review article to this special issue on ECM, and provided a general overview of studies linking the ECM to brain function during development and adulthood. They have discussed and tabulated the effect of stress on both diffuse and structured ECM, and its functional consequences on emotion processing, learning and memory.

In addition to neurodevelopmental disorders, the functional deficit of neural networks following injury can result from poor regeneration of damaged axons and insufficient target innervation due to faulty ECM. Using a rat contusion model of severe spinal cord injury Kabdesh et al. investigated the spatial and temporal changes in the neuron-glial antigen 2 (NG2) proteoglycan. They identified the change in NG2 expressing cell numbers and the molecular shifts in the ECM of the areas distant from the injury site potentially affecting extended axonal growth and synaptic condition. Through their original research article, they reported elevation in NG2 levels in the segments surrounding the injury along with its comprehensive characterization and distribution around the boundaries of scar formation.

Another original research article on ECM proteoglycans, Brevican, and Neurocan by Hußler et al. measured the levels of these signature proteoglycans in the cerebrospinal fluid (CSF) and serum of 96 neurological patients including ALS, epilepsy and small vessel disease cases. They concluded that monitoring the proteolytic cleavage products of brain-derived perineuronal ECM molecules, such as neurocan fragments, may allow insights into the integrity of the brain's extracellular environment and assist as fluid biomarkers for neurological disorders. With more precise detection techniques, and advancement of methods to restore and maintain myelin functions at early disease states hold great potential for alleviating neurodegeneration. White matter abnormalities due to myelin damage have been associated with multiple neurodegenerative conditions. The original research article by Abi-Ghanem et al. examined the selective homing of cysteine-alanine-lysine glutamine (CAQK), tetrapeptide to sites of myelin damage in three different mouse models of acute, immune-mediated, and toxic demyelination. They assessed the homing by administering fluorescein amine (FAM)-labeled CAQK peptides into the bloodstream of mice and analyzing the sites of demyelination in comparison with healthy brain or spinal cord tissue. The labeled peptides were primarily associated with the fibrous ECM deposited in interstitial spaces proximal to reactive astrocytes at the lesion sites. Thus, CAQK peptide targeting can be developed as diagnostic and therapeutic tool aimed at localized myelin repair in multiple sclerosis and associated disorders.

Further with an interest to identify druggable therapeutic targets for neurodegenerative diseases, Moretto et al. presented an overview of the documented roles of ECM components in the spreading of pathological protein aggregates and recognized the lacunas in the field which needs to be addressed. This review underscored the requirement of extensive *in vivo* experiments in animal models of neurodegeneration, which retain the complex ECM web in its native state, and improved cell culture systems to better recapitulate the ECM under neurodegeneration.

Glial cells, predominantly astrocytes, are one of the major sources of ECM molecules during development and in the adult CNS, hence they play a critical role in ECM mediated brain functions. They also release cleavage proteases, the main effectors of ECM and PNN remodeling during development, adulthood, aging, and diseases. The review article by Tewari et al. discussed the recent advances and understandings, how glial cells are central to ECM and PNN remodeling in normal and disease states of the brain. The pivotal contribution of glial cells to the ECM remodeling process encourages further discussion of glia-centric approaches in addition to the focus on neurons for effective treatment modalities.

Taken together, the insightful review and original research articles complied in this Research Topic on the role of extra cellular matrix in neurodevelopment and neurodegeneration will improve our understanding and stimulate further research ideas on ECM. Further research is warranted to explore the potential of ECM as a diagnostic biomarker and therapeutic target for neurodevelopmental disorders and neurodegenerative diseases.

## Author contributions

All authors listed have made a substantial, direct, and intellectual contribution to the work and approved it for publication.
